# Supplementary material for: Is publication bias present in gastroenterological research? An analysis of abstracts presented at an annual congress
Source: PeerJ. 2018 Jun 22;6:e4995. doi: 10.7717/peerj.4995 (PMC6016530; doi:10.7717/peerj.4995)
Supplement: Appendix 1 [file peerj-06-4995-s002.docx]

Appendix 1: Email sent to authors whose article could not be found as published

Dear Dr.

We are conducting a study to evaluate the rates of publication of podium and poster presentations at the AHPBA Annual Meetings. We have you listed as an author on the following presentation(s):

To make our study results as complete as possible, we would like to know whether this study has been published.  In the event that your conference presentation was not published, we would also like to know the reason for non-publication.  We would appreciate your response to the following questions.

Was this conference presentation published?

If so, please provide the reference information, including authors’ names, title, journal, and year of publication.

If **NOT** published, please provide the **reason** for non-publication from the list below:

Lack of time

Low priority

Lack of funding

Lack of manpower

Study incomplete or ongoing

Publication not an aim

Author/Co-author problems

Poor study quality or design

Fear/Expectation of journal rejection

Results not important enough

Results negative

Similar findings published previously

Sponsor/Funder problems

Publication not permitted by sponsor

In preparation or under review

Initial submission rejected, no intention of resubmission

Inadequate sample size/recruitment problems

Study terminated due to adverse events/toxicity

Other

Thank you for your cooperation and assistance in completing our study.

Sincerely,
